# Debating coffee and hypertension: coffee may increase the risk for chronic hypertension

**DOI:** 10.1093/ajh/hpag017

**Published:** 2026-04-19

**Authors:** Paolo Palatini

**Affiliations:** Studium Patavinum, Dipartimento di Medicina, University of Padova, Padua, Italy

**Keywords:** coffee, genotype, sympathetic, blood pressure, hypertension

Although acute intake of coffee has been demonstrated to raise blood pressure (BP),^1^^,^^2^ adaptation to chronic coffee consumption seems to occur, and tolerance to the cardiovascular responses has been reported.^3^ According to a meta-analysis of short-term trials, the BP increase with a median intake of five cups/day is modest (2.4 mmHg increase for systolic BP and 1.2 mmHg increase for diastolic BP).^4^ Different effects between acute and chronic coffee consumption have been found also for other outcomes. In a recent study, Marcus et al found no relationship between acute coffee intake and premature atrial contractions^5^ whereas in the UK Biobank habitual coffee consumption was associated with less atrial arrhythmias.^6^ Discrepant results were observed also for glucose metabolism. Despite epidemiologic evidence that coffee drinkers have a lower risk of diabetes than persons who do not drink coffee,^7^ in the Marcus et al study^5^ there was no evidence that short-term coffee consumption was associated with mean daily glucose levels.

Longitudinal prospective studies can clarify whether the pressor effect of coffee use seen in short-term clinical trials translates into an increased risk of developing hypertension over time. In 1107 participants from the Hypertension and Ambulatory Recording Venetia Study (HARVEST), a prospective longitudinal study of young individuals screened for stage 1 hypertension, followed for 6.4 years, coffee drinkers developed sustained hypertension more frequently than abstainers (53.1% vs. 43.9%, *P* = .007).^8^ In the HARVEST, participants were grouped into three categories of coffee drinking, non drinkers (none), moderate drinkers (1-3 cups/day) and heavy drinkers (4 or more cups/day). The adjusted relative risk of hypertension was greater in both categories of coffee drinking than in abstainers. In a more recent analysis of the HARVEST study in 1206 participants, the independent risk of hypertension related to coffee intake (yes versus no) in an 11.5-year follow-up was 1.24 (1.09-1.42, *P* = .001).^9^ However, conflicting results have been reported in other studies.

In a meta-analysis of 13 cohort studies of chronic coffee consumption, a null association with hypertension was found.^10^ In contrast, a more recent meta-analysis showed that high coffee consumption was associated with a 7% reduction in the risk of hypertension.^11^ However, the most intriguing finding was that the results varied by study characteristics, such as the region of study, study quality, etc. This suggests that when studying the longitudinal effects of coffee on BP and other clinical variables, its effects should be investigated on specific subgroups, as coffee might be detrimental in some people and neutral or even beneficial in some others. In this review the relationship between coffee drinking and risk of hypertension will be considered not only within the general population but also among individuals stratified according to their genetic characteristics and physiological features.

## Effects of coffee according to genetic background

It has been shown that over 95% of caffeine is metabolized by cytochrome P450 1A2 (CYP1A2) and that a common polymorphism in the *CYP1A2* gene affects caffeine metabolism.^12^ The genetic variant rs762551 decreases enzyme activity and inducibility. Carriers of the C allele (*1F allele) are considered as “slow” metabolizers, and those with the AA genotype (*1A/*1A) as “fast” metabolizers. This polymorphism has been shown to affect the association of coffee intake with risk of myocardial infarction.^13^ In fast metabolizers, increasing cups of coffee consumed per day was associated with either a lower risk or no association with myocardial infarction, whereas in slow metabolizers, an increased risk was observed. A similar analysis was conducted by our group within the HARVEST study to investigate the effect of coffee intake on the risk of developing hypertension needing antihypertensive treatment.^14^ In 553 individuals stratified by CYP1A2 genotype, we assessed prospectively the risk of new-onset established hypertension. During a median follow-up of 8.2 years, incident hypertension was diagnosed in 323 individuals. For carriers of the slow *1F allele (59%), hazard ratios of hypertension from multivariable Cox analysis were 1.00 in coffee abstainers (reference group), 1.72 (95% CI, 1.21-2.44) in moderate coffee drinkers (*P* = .03), and 3.00 (1.53-5.90) in heavy drinkers (*P* = .001). In contrast, hazard ratios for coffee drinkers with the rapid *1A/*1A genotype were 0.80 (0.52-1.23, *P* = .29) for moderate drinkers and 0.36 (0.14-0.89, *P* = .026) for heavy drinkers. Twenty-four-hour urinary epinephrine was higher in coffee drinkers than abstainers but only among individuals with slow *1F allele (*P* = .001). In addition, heavy coffee drinkers carriers of the slow *1F allele had a higher adjusted risk of prediabetes (HR, 2.8, 95% CI, 1.3-5.9) compared to abstainers, whereas this association was not significant among the homozygous for the A allele. These data show that coffee consumption increases the risk of hypertension and impaired fasting glucose only among carriers of the slow CYP1A2 *1F allele.

Caffeine can exert adverse effects on renal structure and function by stimulating some of the proliferative mechanisms involved in glomerular remodeling and sclerosis. In the HARVEST, risks of developing albuminuria and hyperfiltration, assessed by Cox regression and survival analyses, were not related to coffee intake when the entire group was taken into account.^15^ However, the risk of albuminuria and hyperfiltration increased with heavy coffee intake among the participants with the C allele of CYP1A2 rs762551, associated with slow caffeine metabolism, suggesting that in susceptible subjects caffeine may play a role in the development of renal dysfunction.^15^

## Coffee effects according to sympatho-adrenergic activity

Young people in the early stage of hypertension often exhibit hyperkinetic circulation and other signs of increased sympathetic tone. It is thus conceivable that caffeine, a chemical that causes a further increase in sympatho-adrenergic activity and promotes adrenal medullary release of epinephrine in response to stress,^16^ may have detrimental cardiovascular effects in these subjects. A recent analysis in the subgroup of HARVEST participants who underwent 24-hour urinary catecholamine assessment (*N* = 630) showed that the risk of hypertension associated with coffee intake greatly differed according to sympatho-adrenergic activity (unpublished results). In a multivariable Cox analysis, coffee consumption was associated with hypertension only among the participants with high sympatho-adrenergic tone, especially in those who drank more than 3 cups/day (hazard ratio, 2.60 (1.57-4.28) for norepinephrine and 1.83, (1.11-3.04) for epinephrine) while no increase in risk was found in those with low sympatho-adrenergic tone (*P* = n.s. for both). The cardiovascular effects of coffee can be further amplified by smoking which increases neural sympathetic outflow to blood vessels.^17^

## CV Effects of coffee in patients with hypertension

**Figure 1:**
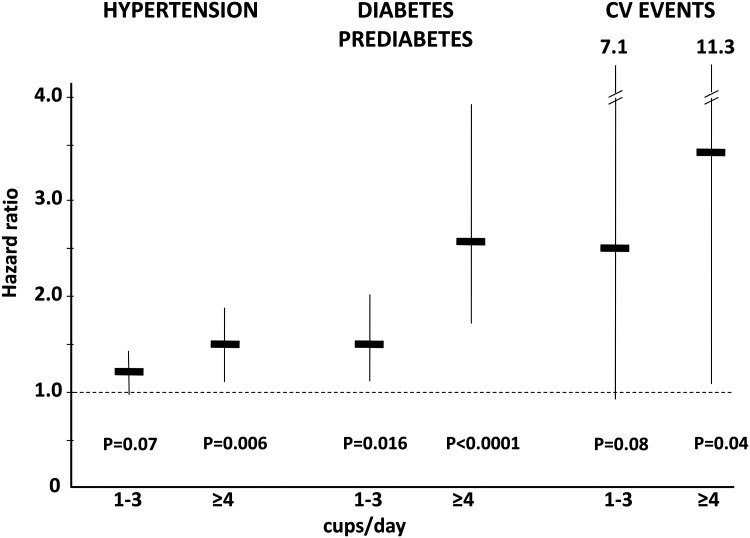
Risk of hypertension, diabetes/prediabetes, and cardiovascular events according to coffee consumption (cups/day) during a median follow-up of 12.6 years in 1204 HARVEST participants. Coffee abstainers were considered as the reference group. The risk (hazard ratio) was calculated from a multivariable cox regression including participants’ demographics and other risk factors. Adapted from Palatini P et al., *Int J Cardiol* 2016;212:131.

Controversy still exists about the long-term cardiovascular effects of coffee consumption in patients with hypertension. Most of the previous studies were performed in general populations, and results of recent meta-analyses suggest that coffee consumption is safe in people without hypertension free of cardiac diseases. Much less is known about the cardiovascular effects of coffee in patients with hypertension. In a study by Hakim et al^18^ in 499 men, a linear relationship was found between coffee intake and risk of thromboembolic stroke, which was more than doubled in the participants who consumed 3 cups of coffee/day. However, other studies performed in cohorts with hypertension or general populations in which a sub-cohort developed hypertension during follow-up did not confirm those findings.^19^^,^^20^ In the HARVEST study of 1204 young-to-middle-age participants, followed for 12.6 years, coffee use was associated with cardiovascular events, with a hazard ratio of 2.8 in moderate coffee drinkers and of 4.5 in heavy drinkers compared to abstainers (Figure 1).^21^ In keeping with these results, another Italian study performed in a large population found that consumption of over 2 cups/day of espresso coffee was linearly associated with increased risk of coronary artery disease.^22^

## Can cardiovascular effects of coffee vary according to brew preparation?

This stands as one of the most debatable topics among coffee investigators today, as no comparative studies have been conducted within large populations. However, it is noteworthy to observe that in the meta-analysis by Haghighatdoost et al,^11^ when studies were stratified based on the geographical region, higher consumption of coffee was associated with lower risk of hypertension in studies that were conducted in the United States, whereas no such association was observed in Europe or Asia. This difference may be due to the different type of coffee consumed in different countries. In the United States, filtered coffee is the most consumed brew, whereas in several European countries, the most popular coffee preparation is boiled coffee or espresso in which coffee is brewed by passing hot water pressurized by steam through ground coffee. Diterpenes are extracted by hot water but are retained by a paper filter. This explains why filtered coffee does not affect cholesterol compared to boiled coffee or espresso, which have higher concentrations of diterpenes.^23^ In a recent analysis of the Biobank database, a U-shaped association between unsweetened coffee consumption and new-onset hypertension was documented, regardless of coffee type or the addition of milk to coffee.^24^

## Conclusions

Although coffee can be safely consumed by most healthy adults and might even have beneficial effects in some individuals, it should be borne in mind that the cardiovascular effects of coffee may have detrimental effects in potentially susceptible subgroups. The risk of hypertension, metabolic abnormalities, and cardiovascular events may be increased in coffee drinkers who are carriers of the slow CYP1A2 *1F allele and in subjects with enhanced sympatho-adrenergic tone, a susceptibility exacerbated by smoking. Coffee effects on the cardiovascular system may also vary according to the type of coffee brew consumed, which can affect its chemical composition, but no systematic studies have been conducted so far to clarify this issue. Finally, some studies suggest that high coffee intake is linked to cardiovascular disease in patients with hypertension. Thus, potential adverse effects associated with heavy coffee consumption should be considered in the people with hypertension, especially in susceptible categories. The above findings support the use of population-specific, genotype-guided strategies that could contribute to more effective prevention of hypertension and its cardiovascular consequences.
